# Artificial intelligence meets brain theory (again)

**DOI:** 10.1007/s00422-025-01013-5

**Published:** 2025-06-28

**Authors:** Michael A. Arbib

**Affiliations:** University of California at San Diego, La Jolla, CA USA

**Keywords:** NeuroAI, Artificial intelligence, Brain theory, Cybernetics, Neuromorphic computing, Human-machine symbiosis

## Abstract

After noting the cybernetic origins of *Kybernetik*/ *Biological Cybernetics*, we respond to the Editorial by Fellous et al. (2025) and then analyze talks from the NIH BRAIN NeuroAI 2024 Workshop to get one “snapshot” of the state of the conversation between Artificial intelligence (AI) and brain theory (BT). Key recommendations going beyond the earlier Editorial are that: (i) Successes in fitting ANNs to increasingly large neuroscience datasets must not distract us from the quixotic but demanding quest to understand “how the brain works” and discover underlying brain (and AI) operating principles. (ii) We must integrate functional and structural analyses in exploring systems of systems, integrating structures (e.g., brain regions, cortical modules) and functions (e.g., schemas for perception, action and cognition) that bridge between neural circuitry and patterns of behavior. (iii) We must study the diversity of intelligences exhibited by animals in their strategies for survival and not only the disembodied employment of language and reasoning. Finally and briefly, we note the urgency of assessing the societal implications of an age of increasingly pervasive human-machine symbiosis.

## Cybernetic foundations

*Kybernetik* was founded in 1961 as “A journal dealing with the transmission and processing of information as well as with control processes in both organisms and automata,” a clear nod to Wiener’s “Cybernetics: or Control and Communication in the Animal and the Machine” (Wiener 1948). In Volume 1 of *Kybernetik*, Werner Reichardt (1924–1992),^1^ its founder and Editor-in-Chief, studied the correction process in the Limulus eye quantitatively and in part using an analog computer (Reichardt 1961), while in Volume 2 he reported on optomotor reactions of the fly (Fermi and Reichardt 1963). Thus, the study of how sensorimotor integration and control in both organisms (including humans) and automata is informed by comparative and computational studies was established by the founder of the journal that became *Biological Cybernetics* in 1975. The subtitle of my book *An Introduction to Cybernetics as Artificial Intelligence and Brain Theory* (Arbib 1972b) justifies the “Again” in the title of this perspective.

## A short commentary on Fellous, Thomas, Tiesinga, and Lindner

In a recent Editorial for *Biological Cybernetics*, Fellous, Thomas, Tiesinga, and Lindner (2025) primarily address “What Computational Neuroscience can do for AI” rather than “What AI can do for neuroscience,” whereas a recent NeuroAI meeting (§ 3) has the opposite emphasis. This section comments briefly on the six points of the Editorial, summarizing each point in italics.

E1. *The data centers providing the physical infrastructure for modern artificial intelligence require vast amounts of energy, water, and other resources, whereas human and animal intelligence require only the resources of a living individual.*

Running an *AI utility* for many millions of users consumes vastly more energy than a single human brain. The issue, perhaps, is how to get the per person consumption of energy down to an appropriate level– and contrast the notion of an AI utility with the “artificial brain(s)” of an *AI agent* such as a personal assistant or a robot. Success of “the new AI” currently rests on the use of GPUs to support the massively parallel computations of adaptive ANNs, rather than on using, e.g., neuromorphic computers (see § 3.1) that employ chips specifically structured to exploit neuron-like analog computation (Mead 1989).

E2. *The next step beyond neural computation and artificial neural networks may be synaptic computation and artificial synaptic networks.*

Backpropagation has been a crucial learning mechanism for adjusting synaptic weights in the new AI and in computational models of biological neural networks, but is non-biological. Will the new AI computations invoke *artificial synaptic networks* or find technological “shortcuts” to massively parallel adaptation and processing? § 3.2 demonstrates that synaptic details are but one of the refinements relevant to some studies in BT and possibly AI.

E3. *It is time to build neurally-inspired perceptual, decision-making and navigational algorithms that use emotional processing intrinsically.*

*Action* is a crucial capacity of both animals (including humans) and robots ignored in the Editorial despite its central role in the study of feedback and goal-directed behavior in cybernetics. As for emotion, there are cases in which endowing an AI system with emotion may be irrelevant or even harmful (Arbib 2005a). In some cases (e.g., a tutoring system), simulation of certain emotions at the AI-human interface may increase the usability of the AI system. To assess in what sense a particular AI system should itself be endowed with “emotions” will need to build on the psychololgy and neurobiology– from neuromodulation to social interaction– and robotics of emotion (Fellous and Arbib 2005 offers early reviews).

E4. *It may be time to redesign the current approaches to intrinsically include explainability and trustworthiness.*

Humans are neither explainable nor trustworthy. At the NeuroAI meeting (the focus of § 3), Mineault asserted that the human brain is currently the only safe general intelligence -- but human history and current events are replete with examples of human “intelligence” that are unsafe.

E5. *Can AI algorithms and architectures use the deeper insights that are still being obtained from the brains of insects or mammals?*

There may indeed be fruitful interaction between, for example, the study of animal behavior and the development of “brains”, bodies (including sensors and effectors), and action-oriented perception, as in low-level vision or robotics. However, LLMs are very different from biological brains.

E6. *The brain is a massively parallel device, tolerating a massively large amount of apparent noise. Yet, its computations can be exquisitely precise and reliable. Thus noise is a feature of the system, not merely a bug to be compensated for by redundancy.*

Noise (or at least chaos) may enable an animal’s escape path from a predator to be unpredictable, while the small probability of exploration rather than exploitation of an apparently successful protocol is a key to reinforcement learning.

## The 2024 NIH BRAIN neuroai workshop

The NIH BRAIN NeuroAI 2024 Workshop (held on November 12 and 13) offered a partial view of the AI↔BT relationship. Around half the talks (Table 1) are discussed here, with the order changed to improve narrative flow. Brief notes on what a speaker said are indented. No one meeting can cover all aspects of AI↔BT, but the lacunae noted below provide useful entry points for the AI↔BT conversation.

**Table 1:** Selected talks from the NIH BRAIN NeuroAI 2024 workshop in order of presentation at the meeting

Speaker Name	Title of Talk	Primary Theme for § 3
**Day 1**		
Wolfgang Losert	Biocomputing with Astrocytes	Subneural computation & learning
Patrick Mineault	Closing the loop between neuroscience and with virtual neuroscience	Mammalian cortex & digital twins
Andreas Tolias	A Less Artificial Intelligence	Mammalian cortex & digital twins
Anton Arkhipov	Bio-realistic modeling of brain circuits	Mammalian cortex & digital twins
Karen S. Rommelfanger	NeuroAI Ethics: A proactive approach for the next 5–10 years (and beyond)	Societal Implications
Doris Tsao	Fulfilling the potential of NeuroAI	Mammalian cortex and digital twins
Panayiota Poirazi	Towards advanced NeuroAI systems with dendrites	Subneural computation and learning
Carina Curto	The future of NeuroAI: inspiration from insects and mathematics	Mathematical modeling
Evelina Fedorenko	Neural network language models as models of language processing in the human brain	Language
**Day 2**		
Brad Aimone	How Neuromorphic Computing Can Help Us Understand the Brain	Neuromorphic Computing
Kwabena Boahen	Scaling Knowledge Processing from 2D Chips to 3D Brains	Neuromorphic Computing
SueYeon Chung	Learning from Neural Manifolds: From Biological Efficiency to Engineered Intelligence	Mathematical modeling
Dhireesha Kudithipudi	A Co-Design Approach to Continual Learning: Exploring Synergies Between Neuroscience and Neuromorphic Hardware	Subneural computation and learning
Mitra Hartmann	Embodied intelligence through the integration of biomechanics and neuroscience	Biomechanics and motor control
William Nourse	Towards Insect-Scale Intelligence for Robotics	Subneural computation and learning
Kai Miller	Artificial Intelligence and the Functional Neurosurgeon	Biomedical Applications
Giacomo Indiveri	Mixed-signal neuromorphic Systems for next-generation Brain-Computer Interfaces	Biomedical Applications
Ralph Etienne-Cummings	Synthesis of Neuromorphic Principles in Biomedicine and Healthcare Workshop	Neuromorphic Computing

Abstracts for these and other talks are at https://n4solutionsllc.com/brain-program-book/. Videos are at https://videocast.nih.gov/watch=55160 for Day 1, and https://videocast.nih.gov/watch=55262 for Day 2

### Neuromorphic computing

An important theme was the development of neuromorphic circuitry to expand the capabilities and reduce the power demands of neural computations (recall E1).

Brad Aimone presented an implementation that supports over a million CMOS neurons and billions of synapses, roughly the number of the parrot or small primate brain. Neurons are simulated using a leaky integrate-and-fire model with no neuromodulation. In future, “post-Moore devices” (electrochemical random-access memory, memristors, circuits that utilize magnetic tunnel junctions, and various optical and organic devices, etc.) may scale to human sizes.

Aimone also discussed modelling circuitry in Drosophila (see § 3.2).

Kwabena Boahen addressed the challenge of scaling processing from 2D chips to 3D brains and outlined an approach to neuromorphic computation based on considering the brain’s fundamental unit of computation to be dendrocentric learning with sequence detectors (Boahen 2022).

Indeed, much complexity of computation in biological neurons is mediated by the arrangement of inputs on different parts of the dendritic tree (see Poirazi in § 3.2), an important aspect of subneural neuromorphic computation.

Ralph Etienne-Cummings reported on the 2024 Workshop on Neuromorphic Principles in Biomedicine and Healthcare, addressing challenges of creating a new generation of biomedical and neurotechnologies that operate with extreme energy and data efficiency, adaptability, and performance advantages. They also noted the need for new electronics materials that better interface with tissue and possess stretchability and conformability, biocompatibility, self-healing capabilities, and low immune response. In some sense, the task involves reverse engineering the brain (Cauwenberghs 2013), but *speakers cautioned against too strict an adherence to biological mimicry*,* suggesting a broader “physiomorphic” framework*. [My italics]

Different properties are relevant for implantable or wearable neuromorphic circuitry as a tool for healthcare from those for free-standing AI agents. A basic question reiterated in § 4.2 is “at what level should the ‘neural circuit equivalent’ be designed or adapt”?

### Subneural computation and learning

Diverse areas of the human brain employ different forms of synaptic plasticity (recall E2), and the brain comprises more than neurons. BT relates diverse forms of neuromodulation and synaptic plasticity to the role of different brain regions in supporting different “psychological-level” styles of learning and memory (Caligiore et al. 2019; Doya 2000) including episodic memory (based in part on the hippocampus), procedural memory (integrating cerebral, cerebellar, midbrain and even spinal mechanisms) and reinforcement learning (where the role of dopamine in learning within the basal ganglia is the classic example). Lifelong learning provides a related challenge:

Dhireesha Kudithipudi stressed the importance of machines that exhibit lifelong learning without catastrophic forgetting (van de Ven et al. 2024). In synaptic consolidation, newly formed synaptic connections become stable and integrated into the network over time, making the synapses more enduring and less prone to disruption. He argued that neuromorphic hardware that integrates probabilistic switching and the inherent variability of non-volatile memory can represent plasticity mechanisms through fine-grained reconfigurability units within the memory.

Kudithipudi’s talk suggests the importance of deeper analysis of diverse memory mechanisms even at the cognitive level as a target for AI-BT cooperation in shaping neural computing. Episodic memory encodes new episodes in which the agent is involved. One hypothesis is that some of these memories first formed in hippocampus become consolidated in neocortical circuits (McClelland et al. 1995; Squire and Alvarez 1995). Another, is that an increasing stock of episodic memories is supported in part by neurogenesis in the hippocampus (Aimone et al. 2009), suggesting that episodic memory might demand the use of growing circuitry. Could neuromorphic computing address this other than by deploying larger and larger portions of a prestructured network? For semantic memory, the registration of contradictions may initiate overwriting of one “fact” by “another”. In procedural learning not only can new skills be mastered, but old skills can be honed by continuing practice– a long-standing application area for applying ANNs to adaptive motor control (Albus 1975).

Two speakers moved beyond E2’s concern with artificial synaptic networks.

Wolfgang Losert introduced biocomputing with astrocytes as carriers of analog information and as enablers of slow integrative processing of information in neural networks.

This talk asks us whether one should consider “neuron + astrocyte” as a unit for biological NNs.

Panayiota Poirazi reviewed specific dendritic structures that empower brain function. They not only segregate neuronal inputs to a neuron and thus support differential plasticity of existing synapses but also support where new synapses may form, e.g., in clustering. She then suggested the challenges of extending both the neuroscience and the technology to advance dendrite-inspired computing (Pagkalos et al. 2024).

This suggests that dendritic compartments might become units for neuromorphic computing. Recall the original insights of Rall (1964), and the rich array of biological neural modeling on “conventional” computers using, e.g., NEURON -- and note Boahen’s concern with neuromorphic circuitry that supports dendrocentric learning. The second property of synaptogenesis (and apoptosis) seems (to me) less tractable for neuromorphic computing.

William Nourse offered examples of “insect intelligence” (e.g., cooperative behavior of ants forming a formic bridge to cross a gap) but no analysis of how neural networks might support such behavior. Rather, he focused on the work of Hulse et al. (2021) on “A connectome of the Drosophila central complex reveals network motifs suitable for flexible navigation and context-dependent action selection,” but his example of a “fruit fly robot” was based on fly leg biomechanics (cf. Hartmann, below), not details of the fly nervous system.^2^

Nourse added that there is no complete whole-nervous-system connectome -- elements are missing, such as electrical/gap junctions and fibers between sensory systems and ventral nerve cord. If we had them, would it make it easier to model the collective behaviors of insects? Ant bridge building or termite mound building (Turner 2011) can be understood without invoking neural circuitry but by invoking relatively simple rules as studied in “swarm intelligence.”

Aimone also mentioned that Shiu et al. (2024) exploit the recent central brain connectome of adult *Drosophila melanogaster*, containing more than 125,000 neurons and 50 million synaptic connections to model the entire Drosophila brain on the basis of neural connectivity and neurotransmitter identity. It accurately models the activation of gustatory neurons and of a small set of neurons comprising the antennal grooming circuit. Aimone claims that the brain likely only computes what the brain is efficient at computing, and that neuromorphics should have similar restrictions.

Will more expressive neuromorphic circuitry involve incorporating more and more subneural mechanisms (compare the synaptic networks of E2 and the dendritic computing just discussed)? Note that certain neurons (whether in Drosophila or human) are so complex that the circuitry required for the simulation of one such neuron may prove as complex as the circuitry now used to simulate many current artificial neurons. Alternatively, will they gain economy by discovering places where instead of representing a single neuron, a subcircuit can represent a pool of neurons or larger structural elements like columns (Hubel et al. 1978; Mountcastle 1997; Powell and Mountcastle 1959) or functional units like *schemas* (as explained in T2 of § 4).

Moreover, if neuromorphics should only compute what the brain is efficient at computing, what are we to learn from the neural architecture of Drosophila versus the very different architecture of different regions within a mammalian brain. Conversely, consider the way the human brain supports diverse “virtual machines.” The combination of cultural evolution and development equips humans to do many things the brain did not originally evolve for. Modeling all connectome-specified neural connections is very different from starting with a “standard” layered (possibly recurrent) ANN.

Between them, these talks raise the issue of when and whether “we” need all the details of biological neurons, whether in an AI application or in modeling a particular set of phenomena in BT. When we do, what biological details should be included?

### Mammalian cortex and digital twins

With this we turn to simulations of systems akin to regions of mammalian cortex.

Patrick Mineault proposed developing digital twins and foundation models that enable in silico experimentation and hypothesis generation to better understand perception, cognition, and behavior.

Andreas Tolias notes the new neurotechnologies that now allow neurophysiologists to collect brain data at unprecedented scale and precision, while AI provides tools that can ingest vast, complex data to make predictions. With this, digital twins of the brain can support unlimited in silico experiments and the application of AI interpretability tools, enhancing our understanding of neural computations.

The term “digital twins” here seems to be simply a relabelling of the well-established notion of a computational model of some aspect of brain function. Disturbingly, previous work on modelling the diversity of brain systems was substantially ignored in this meeting. Moreover, approximating the task-dependent behaviour of a region using an ANN whose structure is unconstrained seems misleading when the goal is to use BT to advance our understanding of brain structure and function -- though more is being learned when the output being considered include data on the firing patterns of neurons during certain tasks.

Anton Arkhipov argued that the next decade will combine dense reconstructions of the circuitry and neural activity across whole brains (in the mouse) or large portions of the brain (in non-human primate and human brain tissue) with bio-realistic modeling of these reconstructed circuits. Experimentally, this will leverage electron and light microscopy, expansion microscopy, spatial transcriptomics, large-scale optical- and electrophysiology, and associated AI tools.

The wealth of new methods Arkhipov mentions will certainly open up new findings in neuroscience. But the map is not the territory (Borges 1975). If we develop “overly isomorphic” digital twins, no matter how well trained by AI/ML techniques, have we gained understanding of “how the brain works”? See § 4.2.

Doris Tsao studied activity of macaque neurons in inferotemporal cortex during face recognition and discovered that cells in patches represent facial identity along two “axes” coding shape (an “axis” with 25 coordinates) and appearance (the other “axis,” 25 coordinates).

Tsao’s work on inferotemporal cortex, though limited to a very specific task of the visual system, offered the meeting’s most impressive demonstration of linking neurotechnology and machine learning to make progress in neuroscience. Moreover, she was the only speaker to stress the importance of insights from cognitive science, briefly citing concepts for vision science of surface representation, object files, and the recognition of equivalence under, for example, rotation.

### Mathematical modeling

A different path to understanding the brain is offered by mathematical results that offer qualitative analyses of neurobehavioral phenomena– while laying the foundation for computational models that can address numerical details.

Carina Curto noted that without the notions of eigenvectors and eigenvalues for linear systems, many insights into system performance would be unobtainable. We cannot expect a similar handle for nonlinear systems in general but threshold-linear networks are a promising subclass that could connect graph structure and architecture, with the example of encoding multiple gaits in a recurrent network.

Threshold-linear networks were developed, for example, by Shun-Ichi Amari and applied to winner-take-all networks and stereopsis in an early paper on dynamic fields (Amari and Arbib 1977).

SueYeon Chung argued that deciphering geometry of neural manifolds and its computational role is crucial to understanding the emergence of intelligence by supplying key metrics for interpreting the structure of internal representations of the brain and Al systems. Studies briefly cited included the geometry of manifolds for deep neural networks for vision, for hierarchy in language, for invariant speech recognition, as well as manifolds related to mouse hippocampus (navigation), monkey motor cortex (reaching), and even the geometry of social learning.

Chung’s application of the notion of neural manifolds to model various specific systems is important, but we need to better understand how these new models relate to earlier models and whether the methodology does extend to support the “emergence of intelligence.” Crucially, though, mathematical results may suggest constraints on the structure of digital twins, specifying parameters for which machine learning may offer new insights.

### Biomechanics and motor control

Action and motor control are highly relevant to BT since brains evolved to enable animals to survive by perceiving the world not as an end in itself but to enable choices as to what to do next while building up memories that might guide future action, Some attention was paid to the visual systems of flies and primates but motor control was almost completely ignored in the Workshop. One of the few exceptions was Hartmann’s:

Mitra Hartmann stressed that understanding neural function in organisms will ultimately require integrating accurate biomechanical models of sensors and muscles with neurophysiological data. She exemplified this in her study of rodent whisking, a system that operates in service of perceiving the environment. She paid particular attention to the details of neural innervation of each whisker, and the biomechanics of the whisker and its associated musculature. Her study of the biomechanical sensor was complemented by a model of sensing shape by whisking.

Rodents using whisking to perceive the shape of the environment have long been a target for studies of both animal behavior (ethology) and robot design (Mitchinson and Prescott 2013; Prescott and Wilson 2023). Such studies raise the issue of which mechanoreceptor details really matter for NeuroAI or robots, as distinct from neurobiology. Again, we see the tension between “understanding brains” and “building machines that can emulate (*more or less*) a ‘useful’ brain function,” whether or not AI is involved.

### Intelligence and language

Surprisingly for a meeting with a concern for AI, none of the talks analyzed the notion of intelligence, and how it might best be understood for non-human animals and machines. Nourse spoke of “insect intelligence” but failed to define it. The closest the Workshop came to the notion of human intelligence was the discussion of LLMs and language, but no attempt was made to assess how the human brain differs from other brains to make human intelligence distinctive

Evelina Fedorenko sees animal studies as useful in relation to some aspects of human cognition but *not* for language. She defines the *language system* as a network of left frontal and temporal areas in the human brain that supports language by retrieving words from memory and building syntactic structures in the service of semantic composition (Shain et al. 2024). She dissociates it from systems for reasoning and does not include Wernicke’s and Broca’s classical areas for articulation and speech perception because “they are insensitive to what language-like input is given.” The link to AI is that “a new candidate model organism has emerged, albeit not a biological one, for the study of language —large language models (LLMs).” These models exhibit human-level performance on diverse language tasks, and she stated that their internal representations are similar to the representations in the human brain when processing the same linguistic inputs (Schrimpf et al. 2021).

There is no doubt that AI has been greatly influenced by the remarkable performance exhibited by LLMs, but I have reservations about Fedorenko’s program. First, she dismisses the relevance of animal studies to language, whereas much is to be learned by exploring evolutionary pathways from the posited manual abilities of our last common ancestor with the great apes and comparative neuroethology of humans and extant primates (Arbib 2005b, 2020). A different concern is that the complete input sequence (the prompt) to an LLM is maintained in a buffer as the output is produced through iterations that each produce one word with output-so-far stored in its own buffer. No internal working memory is maintained of the overall plan between iterations for what is to be said, though it might be claimed that such information is recomputed at each cycle. Such issues may matter both for more economical AI computation while casting doubt on LLMs as a basis for understanding neural correlates such as the generation of the classic ERP data on language processing.

### Biomedical applications

Ironically for an NIH meeting, there was almost no discussion of the medical relevance of NeuroAI. One exception concerned neurosurgery:

Kai Miller assessed how AI may assist functional neurosurgeons in identifying structure in biological measurements, documentation and chart synthesis, and clinical prediction, and especially in the development of closed-loop devices. For the latter, the aim will be to match the measurement scale of implanted devices to the physical scale of the neurophysiological feature (embodied measurement hardware), and then to implement neurologically-inspired algorithms to match the natural statistics and dynamic variation of brain circuitry (neuromorphic computing). He stresses the difference between treating the symptoms of a disease and addressing the circuit dysfunctions underlying the disease (and here gene therapy and drugs may be complemented by AI-improved brain-machine interfaces) and notes that a clinical need may not be the same thing as what is scientifically compelling.

Miller charts possible roles of machine learning in the cycle of patient care. He suggests the need to describe patients for neurosurgery at a level that relates the current patient to earlier patients with known care and outcome. This points to the possible relevance of LLM-type expert systems to aid diagnosis. Understanding data on brain imaging and ERP may need modeling to probe how such measures relate to the underlying neural circuitry (Arbib et al. 1995; Barrès et al. 2013; Horwitz et al. 2005).

Giacomo Indiveri addressed the limitation of current AI technologies when it comes to brain-machine interfaces, requiring real-time interaction with the nervous system. Since both wearable and implantable neural interfaces need to operate continuously for tasks such as real-time anomaly detection, they require extremely low power consumption. He advocates analog neuromorphic electronic circuits and mixed-signal neuromorphic processing systems to minimize power consumption while engaging in continuous dialog with signals produced by neurons in a living (human) brain.

Note that the machine in a brain-machine interface is *not* a digital twin. Rather it must build an internal model of some aspect of neural function adequate to “hold a conversation” with some portions of the brain to provide signals that will maintain the function within desired limits. To this end, Indiveri uses populations of neurons, averages over space and time, and employs negative feedback, adaptation, and learning mechanisms. His message is that thinking at the level of networks may be more economical (and, I add, more “task-relevant”) than importing all the neuron details.

## What might brain theory and AI teach each other?

Whereas the focus of the NeuroAI meeting was on tools for neuroscience, the mission of *Biological Cybernetics* is to advance the study of (to extend Wiener’s list) 4Cs of computation, communication, coordination and control in both animals and machines, with the subtext of looking for underlying principles. The rise of computer networks and related technologies (including AI) means that we live in an age of *human-machine symbiosis* (Arbib 1976; Licklider 1960) and so must increasingly study how the 4Cs operate *between* humans and machines. The societal implications of this will be noted all too briefly in § 5. The present section will focus on suggestions for research on BT and AI that pays particular attention to the interaction between these fields.

### The quest for understanding

For most of the NeuroAI talks, the focus was on an AI that applies machine learning to detect patterns in large masses of data as a possible source of predictions. It remains a key challenge to constrain these tools to develop models that help us gain an *understanding* of the key interactions that underlie various aspects of behavior and cognition.

The Brain Operation Database (BODB; Arbib, Plangprasopchok, Bonaiuto, & Schuler, 2014; Bonaiuto and Arbib 2016) offered ways to *compare* models on the basis of schematics of their subsystems and connections, the brain operating principles they employed, and the subsets of a set of summaries of empirical data each matched. The notion was that one could then compare the models as a basis for constructing a better model that covered the range of successes of the two prior models. BODB failed because it lacked the tools that AI can now provide, but it seems timely to create BODB Redux to support model development in a way that might complement scoring models on their correlation with a large array of detailed empirical data as in BrainScore (Schrimpf et al. 2018, 2021).

An intriguing question: To what extent will the interaction between AI and BT not only deepen our understanding of brain operating principles but also lead to the discovery of further operating principles shared by diverse AI systems (beyond the power of specific learning strategies)?

### Systems of systems: structural and functional

None of the NeuroAI speakers mentioned the human connectome at the level of brain region connectivity (Sporns 2022) nor probed the fact that the brain is a *system of systems* such as specialized areas of cerebral cortex, basal ganglia, cerebellum, hippocampus, and spinal cord that have very different connectivity, cell types, and morphology and plasticity mechanisms for those cell types. A crucial challenge is to better understand why different regions have such distinctive neural architectures, and what implications this might have for building artificial systems.

Shepherd and Grillner (2017) present canonical microcircuit diagrams for 50 brain regions, examining the integration of structure, function, electrophysiology, pharmacology, brain imaging, and behavior through optogenetics, neurotransmitter uncaging, computational models of neurons and microcircuits, and more. They argue that data from new genetic tools when applied to microcircuits in the mouse and Drosophila allow them to extract common principles across vertebrate and invertebrate microcircuit systems. A complementary question is whether distinct neuromorphic computers must be developed to efficiently provide “digital twins” for each of these 50 brain regions, or whether the common principles could support the development of a single “brain chip” architecture that can be adapted to yield efficient computational models for all these regions. Perhaps neurorobots will require a variety of specialized neuro-chips for different tasks.

A corollary is that neuroscientists in interaction with cognitive scientists must assess how much detail of neural function is needed for different kinds of cognitive analysis (e.g., human-machine coupling in education) and medical therapy. The brains of people or animals in social and physical interaction can be analyzed *structurally* in terms of brain regions, layers, modules, and so on, or *functionally* in terms of networks of schemas. But what are schemas? There are varied versions of schema theory (including those of Bartlett 1932; Head and Holmes 1911; Lakoff and Johnson 1999; Schmidt 1975) but the choice of the name “schema” in my approach (Arbib 1975, 1987, 1992) was inspired by the use of the term in the genetic epistemology of Piaget (1954, 1971). The functional representation by schemas is demonstrably real in terms of cognition and behavior -- without denying that there will be cases in which a subschema or neural network level of analysis may be important. If an animal is to survive in its world, then schemas as dynamic systems for perception, action and cognition (and also linked to motivation and emotion) are crucial to that survival. In particular, the approach singled out perceptual and motor schemas.

A *perceptual schema* allows us to recognize, say, a car or a person or an action and salient details to generate a “schema assemblage” that captures *aspects* of the scene Including the *fine details of affordances* relevant to ongoing behaviour.

A *motor schema* is, basically, a control system for controlling some aspect of that environment.

Many other schemas serve to coordinate such schemas, and support working memory, episodic memory, and procedural memory as appropriate in varied contexts. A crucial aspect of the theory is that motor schemas rely on perceptual schemas to adjust their parameters to the current environment. This is crucial for robotics (Lyons and Arbib 1989) but such coordination may become irrelevant in much of BT and AI where the emphasis is on cognition alone or language rather than the control of behavior.

The demands of occupational therapy may be met at the level of schema theory whereas the treatment of a psychiatric disorder may require therapies informed by genetic analysis of the patient. In modeling a system of systems of the brain, one part may be mapped in detail, while others may be treated in terms of schemas that summarize the “contracts” as to what inputs they provide or how use is made of its outputs. Analysis of fMRI data may stop at the level of relating brain regions and schemas, but other studies may bring the functional and structural analyses together at the level of neural networks.

### What is intelligence?

The quest to develop intelligent machines or, indeed, more fully understand human and animal intelligence was ignored at the Workshop. One approach says that an animal is intelligent if it can master the art of survival and reproduction in a complex environment. The way in which creatures have evolved ever more complex mechanisms to support intelligence in this sense has long been a goal of comparative neurobiology as approached in an evolutionary perspective (Cisek 2022; Kaas 2017; Schneider 2014). However, some people consider only humans to be intelligent because only we produced a Newton, a Beethoven, or an Einstein, and define intelligence as necessarily involving language and reasoning rather than successful behavior as reference points. But each human develops skills in a particular direction, whether gymnastics or woodworking or playing the fiddle. Though none of us possesses “general intelligence,” we do have social structures in which we can develop diverse forms of individual intelligence, modulated by our neurobiology and our social circumstances. To the extent we are individually intelligent “in a uniquely human way,” it is primarily because we develop in a social nexus that exploits language as we develop behavioral skills, and we can only do this because our brains share core features with those of diverse species (Arbib et al. 2025; d’Errico et al. 2025).

Crucially, then, much intelligence resides in a social group of humans and the external memory encoded in their artefacts (tools, documents, buildings, agriculture, etc.). LLMs store a body of data that belongs to the group while omitting detailed linkages that may be required for “true understanding.” But much of the future utility of AI will involve the development of apps and robots whose intelligence is tailored to the demands of a specific range of tasks in a relatively limited range of (physical, virtual, and social) environments. An intriguing challenge, going beyond continual learning, is to distinguish the structuring of episodic memories into autobiographical memory in humans (Arzy and Dafni-Merom 2020; Nelson and Fivush 2020; Suddendorf et al. 2016), the possible analog for individual robots and some other AI agents, and the customer and surveillance records of AI utilities.

## Societal implications

One talk at the NIH meeting discussed the ethical issues of NeuroAI viewed as the application of AI techniques in neuroscience research and clinical applications.

Karen Rommelfanger noted that NeuroAI promises advances in clinical diagnostics, treatment and restoration of neurological disorders as well as having potential for individual and human AI-enabled augmentation. However, since the brain underlies human identity, agency, autonomy, emotion, thought, and overall lived experience, she warned that each context in which we intervene with and explore the brain will have potential ethical implications. She then introduced some proactive ethical considerations for NeuroAI researchers (Robinson et al. 2022).

However, the neuroscience and AI of social interaction remained unaddressed at the NIH meeting. Current work in social neuroscience, social robotics, and human-machine interaction can all contribute to the development not only of NeuroAI (considering humans not only as individual agents but as members of social groups and broader ecosystems) but also AI systems that may augment humans or diminish them. Will AI agents and utlities enrich human experience or reduce more and more humans to become cogs in AI-dominated workplaces or social outcasts?

Now, perhaps more than ever, experts in biological cybernetics, neuroscience, and artificial intelligence should consider the societal implications of their work. These are not issues for scientists and technologists alone but involve the recalibration of capitalism and other social systems to enhance the dignity of all humans, rather than the privileged few. Developing the relevant issues is outside the scope of this paper, but recall the title of Drew McDermott’s (1976) article “Artificial intelligence meets natural stupidity” and Ralph Waldo’s aphorism that “The stupidity of men always invites the insolence of power.” The issue is not just one for the development of AI or the application of neuroscience; it requires a major reorganization of how humans come together in society.

The challenge is to help eight billion humans find meaning in their individual lives (see the Legend of Tralfamadore in Vonnegut 1959) rather than becoming increasingly disposable in the age of “intelligent” machines. Influenced in great part by the neurophysiologist Curt Bell, the implications of neuroscience and AI for human-machine symbiosis have long concerned me (Arbib 1972a, b, Sect.), This is a crucial topic in which we should all engage but, unlike the issues addressed in previous sections, the debates will primarily continue outside the pages of *Biological Cybernetics.*

## Data Availability

No datasets were generated or analysed during the current study.

## Footnotes

1 See https://www.kyb.tuebingen.mpg.de/79635/werner-reichhardt?page=2 for an obituary.

2 Contrast Reichardt’s classic studies of visuomotor coordination in the fly.
